# Decreased Estimated Glomerular Filtration Rate (eGFR), Not Proteinuria, Is Associated with Asymptomatic Intracranial Arterial Stenosis in Chinese General Population

**DOI:** 10.1038/s41598-017-04549-0

**Published:** 2017-07-04

**Authors:** Zhaoxia Li, Jinxin Li, Anxin Wang, Hua Pan, Shouling Wu, Xingquan Zhao

**Affiliations:** 1 Department of Neurology, Beijing Tian Tan Hospital, Capital Medical University, Beijing, China; 2 China National Clinical Research Center for Neurological Diseases, Capital Medical University, Beijing, China; 3 Center of Stroke, Beijing Institute for Brain Disorders, Beijing, China; 4 Beijing Key Laboratory of Translational Medicine for Cerebrovascular Disease, Beijing, China; 5 Department of Epidemiology and Health Statistics, School of Public Health, Capital Medical University, Beijing, China; 6 Department of Cardiology, Kailuan Hospital, Hebei United University, Tangshan, China

## Abstract

The relationship between chronic kidney disease (CKD), which is defined by declined estimated glomerular filtration rate (eGFR) and/or proteinuria, and asymptomatic intracranial arterial stenosis (ICAS) is largely unknown. We conducted a population-based, cross-sectional study by recruiting 5209 participants free of previous stroke, transient ischemic attack and coronary heart disease. eGFR was calculated using the Chronic Kidney Disease Epidemiology Collaboration (CKD-EPI) formula and proteinuria was estimated with urine dipstick. The presence of ICAS was assessed by transcranial color-coded Doppler (TCD). Out of the whole population, 684 (13.1%) participants suffered ICAS. After adjusting for the confounding factors, eGFR < 45 ml/min/m^2^ was an independent risk factor of asymptomatic ICAS (odds ratio [OR], 3.29, 95% confidence interval [CI], 1.67–6.51), but the trend was different between the two groups stratified by the age of 60 (P = 0.01). However, the association between proteinuria and asymptomatic ICAS was not statistically significant. In conclusion, declined eGFR, not proteinuria, is associated with asymptomatic ICAS in Chinese general population, especially in people over 60 years old.

## Introduction

Intracranial arterial stenosis (ICAS) is the result of atherosclerosis that affects large intracranial arteries^1^. ICAS is one of the most common causes of stroke and is associated with a high risk of recurrence, especially in Asia. It has been estimated that 33% to 84% of Asian ischemic stroke patients also suffered symptomatic or asymptomatic ICAS^2^. Additionally, the one-year risk of stroke recurrence in patients with ICAS(≥70%) may be as high as 14% to 23%, even after receiving medication for secondary prevention^3^. Therefore, it is important to screen the risk factors of ICAS and to prevent the progress through early interventions, which is of great significance to lower the risk of stroke.

Chronic kidney disease (CKD), most commonly defined by a reduction of estimated glomerular filtration rate (eGFR) and/or the presence of proteinuria, affects about 10% to 16% of the adult population^4–7^ and may markedly increase the risk of cardiovascular diseases. CKD could accelerate the process of atherosclerosis independently of traditional risk factors like hypertension and diabetes, although the exact mechanisms call for further research^8^. However, the relationship between CKD and asymptomatic ICAS still needs to be determined.

The aim of the cross-sectional study was to investigate the association between eGFR/proteinuria and asymptomatic ICAS in a Chinese general population free of stroke, transient ischemic attack and coronary heart disease.

## Results

A total of 5209 participants (3182 male and 2027 female) were enrolled in the study. The mean eGFR was 91.44 ± 16.90 ml/min/1.73 m^2^. There were 684 (13.13%) participants who suffered asymptomatic ICAS. The prevalence of ICAS was 10.5%, 16.1%, 18.8% and 44.2% with the eGFR declining from above 90 ml/min/m^2^ to below 45 ml/min/m^2^, respectively (P < 0.001).

### Baseline characteristics according to different levels of eGFR

Baseline clinical and biochemical characteristics of participants according to the classification of eGFR levels were shown in Table 1. Compared to those with normal or near-normal eGFR, the group of subjects with lower levels had more elders, former smokers and drinkers, had a higher proportion of hypertension (HBP), diabetes mellitus (DM) and dyslipidemia, had lower income, were more likely to receive a highest education of middle school and were more inactive in physical exercise. Besides, they tended to have higher systolic blood pressure (SBP), diastolic blood pressure (DBP), triglyceride (TG), total cholesterol (TC), body mass index (BMI) and lower high density lipoprotein (HDL) cholesterol. Proteinuria was more likely to be positive in patients with lower eGFR levels (all P < 0.05).

**Table 1:** Clinical characteristics grouped by baseline estimated glomerular filtration rate (eGFR).

	eGFR (ml/min/1.73 m^2^)				P
	≥90 (n = 3064)	60–89 (n = 1900)	45–59 (n = 202)	<45 (n = 43)	
Age (years)	52.4 (45.9–60.0)	53.9 (45.5–66.8)	51.1 (45.0–66.1)	56.6 (50.6–76.9)	<0.001
Female, n (%)	1288 (42.0%)	640 (33.7%)	88 (43.6%)	11 (25.6%)	<0.001
BMI (kg/m^2^)	24.6 (22.5–26.9)	25.0 (23.0–27.4)	25.0 (23.3–26.8)	26.5 (25.4–27.7)	0.49
SBP (mmHg)	130.0 (118.0–140.0)	131.7 (120.7–148.7)	130.0 (120.0–140.8)	140.0 (130.0–160.0)	<0.001
DBP (mmHg)	80.0 (73.3–90.0)	84.7 (80.0–91.3)	84.3 (80.0–91.9)	88.0 (80.0–93.3)	0.01
Total cholesterol, mmol/l	5.02 (4.43–5.70)	4.84 (4.30–5.53)	4.56 (4.10–5.11)	4.97 (4.65–5.54)	<0.001
Triglycerides, mmol/l	1.33 (0.92–2.03)	1.26 (0.94–1.78)	1.18 (0.97–1.49)	1.45 (1.21–3.18)	<0.001
LDL cholesterol, mmol/l	2.60 (2.13–3.06)	2.63 (2.23–3.09)	2.59 (2.23–2.88)	2.57 (2.22–2.92)	0.33
HDL cholesterol, mmol/l	1.62 (1.33–1.96)	1.46 (1.26–1.76)	1.49 (1.27–1.68)	1.37 (1.18–1.66)	<0.001
Current smoking, n (%)	1065 (34.8%)	546 (28.7%)	62 (30.7%)	18 (41.9%)	<0.001
Current drinking, n(%)	1099 (35.9%)	580 (30.5%)	54 (26.7%)	15 (34.9%)	<0.001
Hypertension, n(%)	1304 (42.6%)	1075 (56.6%)	117 (57.9%)	35 (81.4%)	<0.001
Diabetes mellitus, n(%)	362 (11.8%)	238 (12.5%)	31 (15.4%)	9 (20.9%)	0.14
Dyslipidemia, n (%)	1547 (50.5%)	783 (41.2%)	67 (33.2%)	20 (46.5%)	<0.001
Education, n (%)					<0.001
Illiteracy/Primary	255 (8.3%)	340 (17.9%)	47 (23.3%)	7 (16.3%)	
Middle school	1333 (43.5%)	854 (45.0%)	98 (48.5%)	25 (58.1%)	
High school or above	1476 (48.2%)	706 (37.2%)	57 (28.2%)	11 (25.6%)	
Income, n (%)					<0.001
<¥1,000	651 (21.3%)	504 (26.5%)	76 (37.6%)	13 (30.2%)	
¥1,000–3,000	2128 (69.5%)	1176 (61.9%)	110 (54.5%)	26 (60.5%)	
≥¥3,000	285 (9.3%)	220 (11.6%)	16 (7.9%)	4 (9.3%)	
Physical activity, n (%)					<0.001
Inactive	1316 (43.0%)	663 (34.9%)	88 (43.6%)	19 (44.2%)	
Moderately active	785 (25.6%)	461 (24.3%)	50 (24.8%)	11 (25.6%)	
Very active	963 (31.4%)	776 (40.8%)	64 (31.7%)	13 (30.2%)	
Creatinine, μmol/l	65.2 (58.0–72.0)	90.0 (84.0–98.9)	120.0 (117.0–125.1)	159.1 (145.0–175.0)	<0.001
eGFR, ml/min/1.73 m^2^	108.44 (100.49–119.63)	79.54 (72.51–85.22)	53.96 (49.57–56.78)	40.75 (36.06–42.70)	<0.001
Proteinuria, n (%)	59 (1.9%)	45 (2.4%)	10 (5.0%)	6 (14.0%)	<0.001
ICAS, n (%)	321 (10.5%)	306 (16.1%)	38 (18.8%)	19 (44.2%)	<0.001

Values are described as median (Q1–Q3) or number (%). P values are for differences among groups. To convert creatinine from μmol/l to mg/dl, divided by 88.4; to covert cholesterols from mmol/l to mg/dl, multiply by 38.7; to covert triglyceride from mmol/l to mg/dl, multiply by 88.6. BMI: body mass index; SBP: systolic blood pressure; DBP: diastolic blood pressure; LDL: low density lipoprotein; HDL: high density lipoprotein; eGFR: estimated glomerular filtration rate; ICAS: intracranial artery stenosis.

### The correlations between kidney function and asymptomatic ICAS

Table 2 showed the characteristics of subjects with or without asymptomatic ICAS. Men were more susceptible to ICAS. Participants with ICAS were older, more likely to suffer HBP, DM and dyslipidemia, tended to have an education level of middle school, had lower income and took less physical exercise, had higher SBP, DBP, TC, low density lipoprotein (LDL) cholesterol and creatinine, but lower HDL and eGFR (all P < 0.05).

**Table 2:** Clinical characteristics between groups with or without ICAS.

	Without Asymptomatic ICAS (n = 4525)	With Asymptomatic ICAS (n = 684)	P
Female, n (%)	1785 (39.4%)	242 (35.4%)	0.04
Age (years)	51.9 (45.5–60.0)	59.3 (50.7–72.5)	<0.001
BMI (kg/m^2^)	24.7 (22.7–27.0)	24.8 (22.9–27.3)	0.29
SBP (mmHg)	130.0 (119.3–140.0)	140.0 (130.0–157.3)	<0.001
DBP (mmHg)	80.7 (76.7–90.0)	80.7 (77.3–90.0)	0.08
Total cholesterol, mmol/l	4.92 (4.35–5.60)	5.16 (4.51–5.81)	<0.001
Triglycerides, mmol/l	1.30 (0.92–1.94)	1.33 (0.98–1.92)	0.23
LDL cholesterol, mmol/l	2.60 (2.16–3.05)	2.69 (2.20–3.13)	0.15
HDL cholesterol, mmol/l	1.57 (1.30–1.90)	1.53 (1.28–1.84)	0.046
Current smoking, n (%)	1472 (32.5%)	219 (32.0%)	0.79
Current drinking, n (%)	1529 (33.8%)	219 (32.0%)	0.36
Hypertension, n (%)	2051 (45.3%)	476 (69.6%)	<0.001
Diabetes mellitus, n (%)	490 (10.8%)	150 (21.9%)	<0.001
Dyslipidemia, n (%)	2186 (48.3%)	372 (54.4%)	0.003
Education, n (%)			<0.001
Illiteracy/Primary	499 (11.0%)	150 (21.9%)	
Middle school	2032 (44.9%)	278 (40.6%)	
High school or above	1994 (44.1%)	256 (37.4%)	
Income, n (%)			<0.001
<¥1,000	1081 (23.9%)	163 (28.8%)	
¥1,000–3,000	2998 (66.3%)	442 (64.6%)	
≥¥3,000	446 (9.9%)	79 (11.5%)	
Physical activity, n (%)			0.02
Inactive	1826 (40.4%)	260 (38.0%)	
Moderately active	1157 (25.6%)	150 (21.9%)	
Very active	1542 (34.1%)	274 (40.1%)	
Proteinuria, n(%)	95 (2.1%)	25 (3.7%)	0.02
Creatinine, μmol/l	72.0 (61.0–83.5)	73.0 (63.0–87.0)	0.002
eGFR, ml/min/1.73 m^2^	101.47 (85.78–113.89)	99.22 (82.86–111.86)	0.003

Values are described as median (Q1–Q3) or number (%). P values are for differences among groups. To convert creatinine from μmol/l to mg/dl, divided by 88.4; to covert cholesterols from mmol/l to mg/dl, multiply by 38.7; to covert triglyceride from mmol/l to mg/dl, multiply by 88.6. BMI: body mass index; SBP: systolic blood pressure; DBP: diastolic blood pressure; LDL: low density lipoprotein; HDL: high density lipoprotein; eGFR: estimated glomerular filtration rate; ICAS: intracranial artery stenosis.

In the analysis of the association between the level of eGFR and ICAS, the group with an eGFR of no less than 90 ml/min/1.73 m^2^ was considered as the reference group (Table 3). In unadjusted logistic regression analysis, the risk of ICAS increased sharply as the eGFR declined. After adjusting for all the possible confounders, eGFR < 45/ml/min/m^2^ remained to be an independent indicator of asymptomatic ICAS (eGFR 60–89 ml/min/m^2^, OR 1.12, 95% CI 0.92–1.36; eGFR 45–59 ml/min/m^2^, OR 1.23, 95% CI 0.81–1.87; eGFR < 45 ml/min/m^2^, OR 3.29, 95% CI 1.67–6.51; P for trend = 0.01). However, in the multivariate-adjusted logistic regression, the relationship between proteinuria and asymptomatic ICAS was not statistically significant (OR 1.01, 95% CI 0.62–1.65).

**Table 3:** Multivariate-adjusted OR and 95% CI for asymptomatic ICAS according to eGFR and proteinuria.

	Case (n)	Crude OR (95% Cl)	Model 1 OR (95% Cl)	Model 2 OR (95% Cl)
	ICAS (n = 684)			
eGFR (ml/min/1.73 m^2^)				
≥90	321	1.00 (Reference)	1.00 (Reference)	1.00 (Reference)
60–89	306	1.64 (1.39–1.94)	1.09 (0.90–1.32)	1.12 (0.92–1.36)
45–59	38	1.98 (1.36–2.87)	1.26 (0.85–1.86)	1.23 (0.81–1.87)
<45	19	6.77 (3.67–12.49)	3.92 (2.07–7.43)	3.29 (1.67–6.51)
P for trend		<0.001	0.003	0.01
Proteinuria				
Negative	95	1.00 (Reference)	1.00 (Reference)	1.00 (Reference)
Positive	25	1.77 (1.13–2.77)	1.54 (0.97–2.44)	1.01 (0.62–1.65)

ICAS: intracranial artery stenosis; eGFR: estimated glomerular filtration rate; OR: odds ratio; CI: confidence interval. Model 1: adjusted for age and gender. Model 2: adjusted for age, gender, BMI, SBP, TC, TG, LDL cholesterol, HDL cholesterol, current smoking, current drinking, HBP, DM, dyslipidemia, education, income and physical activity.

Further analysis of the interaction effects on the association between eGFR levels and ICAS was conducted and the results were shown in Table 4. There was a significant difference between subjects older than 60 years old and younger ones (P = 0.01). In subjects with the age of over 60, the prevalence of ICAS significantly increased with the eGFR declining (OR 1.33, 95% CI 1.07–1.66). While, in subjects younger than 60, this association was failed to be found (OR = 1.02, 95% CI 0.82–1.26). Other possible interaction factors including gender, HBP, DM and smoking status all had no effects on the association between eGFR and ICAS (P = 0.41, 0.90, 0.98 and 0.35, respectively), although the OR values for some subgroups were significant.

**Table 4:** Multivariate-adjusted OR and 95% CI for asymptomatic ICAS according to eGFR and proteinuria, stratified by gender, age and selected risk factors.

eGFR (ml/min/1.73 m^2^)	≥90	60–89	45–59	<45	P for trend	P for interaction
Gender						0.41
Men	1	0.94 (0.72–1.22)	0.79 (0.45–1.38)	3.38 (1.45–7.86)	<0.001	
Women	1	1.28 (0.91–1.69)	1.67 (0.88–3.16)	1.90 (0.46–7.81)	0.52	
Age						0.01
Age ≥ 60	1	1.55 (1.10–2.19)	1.36 (0.77–2.38)	3.33 (1.34–8.26)	0.01	
Age < 60	1	0.82 (0.62–1.08)	1.18 (0.58–2.39)	4.09 (1.36–12.30)	0.02	
Hypertension						0.90
No	1	0.96 (0.68–1.36)	1.12 (0.51–2.48)	2.21 (0.35–13.89)	0.80	
Yes	1	1.17 (0.92–1.50)	1.29 (0.79–2.11)	3.69 (1.74–7.79)	0.004	
Diabetes						0.98
No	1	1.11 (0.69–1.78)	1.07 (0.40–2.83)	3.03 (0.62–14.77)	0.33	
Yes	1	1.12 (0.90–1.40)	1.30 (0.81–2.06)	3.35 (1.55–7.21)	0.01	
Smoking status						0.35
Never smoker	1	1.09 (0.85–1.41)	1.28 (0.75–2.16)	2.30 (0.91–5.86)	0.12	
Former smoker	1	1.99 (0.87–4.57)	2.21 (0.46–10.62)	2.52 (0.87–6.54)	0.17	
Current smoker	1	0.95 (0.66–1.36)	0.74 (0.33–1.64)	4.35 (1.31–14.49)	0.44	

ICAS: intracranial artery stenosis; eGFR: estimated glomerular filtration rate; OR: odds ratio; CI: confidence interval. Model 1: adjusted for age and gender. Model 2: adjusted for age, gender, BMI, SBP, TC, TG, LDL cholesterol, HDL cholesterol, current smoking, current drinking, HBP, DM, dyslipidemia, education, income and physical activity.

## Discussion

In this study, we found that eGFR < 45 ml/min/m^2^ was associated with asymptomatic ICAS in a Chinese general population, which was independent of traditional cardiovascular risk factors such as diabetic mellitus, hypertension and dyslipidemia. However, the relationship between proteinuria and ICAS was not significant in our dataset. To the best of our knowledge, this is the first community-based study reporting the relationship between CKD and asymptomatic ICAS in Chinese general population.

Previous studies have demonstrated that CKD increased the risk of stroke^9, 10^ and carotid atherosclerosis^11–13^. However, the independent or combined effects of reduced eGFR and proteinuria on ICAS have not been extensively explored. Prior to our study, only Kang *et al*. reported a close association between renal function and ICAS in 2012^14^. They found that higher serum creatinine concentration and lower eGFR were correlated with increased incidence of ICAS. Compared with the previous research, our study has several strengths. Firstly, because of the large sample volume, we were able to perform subanalysis by age and other potential cardiovascular risk factors, which showed that in the group with the age over 60 years old, subjects with poorer kidney function might be more likely to suffer ICAS, however, this tendency was not found in the group with the age under 60. This result of stratification analysis indicated that it was reasonable to screen ICAS in patients with decreased eGFR, especially in older subjects. Secondly, unlike Kang’s research, intracranial arteries including middle cerebral artery (MCA), anterior cerebral artery (ACA), posterior cerebral artery (PCA), terminal internal carotid artery (TICA), ophthalmic artery (OA), internal carotid artery siphon artery (SIPH), vertebra arteries (VA) and basilar artery (BA) were all detected in our study. Although the method of TCD was not accurate enough, but the results could show the whole status of the intracranial arteries and could provide the clues to further evaluate certain probable abnormal arteries. Thirdly, unlike the study by Kang *et al*. in which they chose eGFR ≤ 83.8 mL/min/1.73 m^2^ as a cutoff point for the reference group, we selected the group with eGFR ≥ 90.0 mL/min/1.73 m^2^ as the reference, which was more intuitive to demonstrate the effects of lower eGFR on asymptomatic ICAS. Our results revealed a critical risk of ICAS for those with an eGFR < 45 ml/min/1.73 m^2^. And when we pooled groups with eGFR 45–59 ml/min/1.73 m^2^ and < 45 ml/min/1.73 m^2^ together for multivariate analysis, the association was still significant (OR 1.44, 95% CI 1.03–2.04) but somewhat underestimated.

To our surprise, we did not find the significant association between proteinuria and asymptomatic ICAS. The negative results might be somewhat due to the limited data of proteinuria. In our dataset, there were only 120 participants who had positive proteinuria, of which only 20 suffered ICAS. The insufficient data may result in decreased power of a test, which could partly account for the reasons.

Pathophysiological mechanisms to explain the relationship between CKD and subclinical atherosclerosis are still unclear. It has been acknowledged that the progression of CKD is related to various clinical risk factors such as hypertension, hyperlipidemia, insulin resistance, systemic inflammation thrombotic factors, vascular endothelial growth factor and systemic endothelial dysfunction^15–17^, although which needs to be further confirmed.

Potential limitations of our study should be mentioned. Firstly, a cross-sectional study as ours cannot prove a causal relationship which usually has to be shown in a longitudinal investigation. Secondly, TCD was used to diagnose ICAS without confirmation with magnetic resonance angiography or other forms of angiography. However, TCD is a widely accepted method for screening intracranial occlusive diseases, especially in a general population, and is easily accepted by participants as a non-invasive test with a low cost^18, 19^. We will use other supplementary diagnostic tools to rectify this weakness. Thirdly, undetected arteries were recognized as non-stenotic and this might underestimate the true prevalence of ICAS in the population, especially among older participants. In addition, dipstick was not an accurate and quantitative method to test the proteinuria precisely and it was not sensitive enough to impaired kidney functions in the early stage. However, for large general population and primary screening, this method was appropriate and acceptable.

In conclusion, the results obtained in this community-based study show that decreased estimated glomerular filtration rate (eGFR), not proteinuria, is associated with asymptomatic intracranial arterial stenosis in Chinese general population. eGFR < 45 ml/min/m^2^ is an independent indicator of asymptomatic ICAS, especially in subjects older than 60 years old.

## Methods

### Study design and Participants

Asymptomatic Polyvascular Abnormalities in Community (APAC) study is a community-based, observational, prospective, long-term follow-up study to investigate the epidemiology of asymptomatic polyvascular abnormalities in Chinese adults. The inclusion criterion was as follows: (1) no history of stroke, transient ischemic attack or coronary heart disease at baseline as assessed by a validated questionnaire; and (2) absence of neurologic deficits typical for stroke which was examined by experienced physicians in the APAC study. From June 2010 to June 2011, a sample of 7000 subjects over 40 years old was randomly selected from the large population of the Kailuan study which included a total of 101,510 employees and retirees (81,110 men) of the Kailuan (Group) Co. Ltd, a large coal mine industry located in Tangshan, Hebei Province. After excluding subjects who refused to participate (n = 1148), failed to accomplish the baseline data collection (n = 36) or didn’t meet the above inclusion criterion (n = 376), a total of 5440 participants were eligible and were included in the APAC study^20^. In our study, 231 participants who lacked relative baseline data were excluded. Therefore, 5209 participants including 3182 men and 2027 women were included in this analysis.

All participants or their legal representatives signed the written informed consent and underwent questionnaire assessment, clinical examination, and laboratory assessment. The study was performed in accordance with the guidelines of Helsinki Declaration and was approved by both the Ethics Committee of the Kailuan General Hospital and Beijing Tiantan Hospital.

### Measurement of proteinuria and eGFR

#### Proteinuria

Proteinuria was detected by urine dipstick. Test results were none, trace, 1+, 2+, and 3+. We defined proteinuria as 1+ or greater protein on baseline dipstick urinalysis (H12-MA, DIRUI N-600) read automatically. Proteinuria of women was detected during non-menstrual period.

#### Estimated glomerular filtration rate (eGFR)

Venous blood was collected from participants using vacuum tubes containing EDTA (Ethylene Diamine Tetraacetic Acid) after overnight fast (>8 h). All blood samples were processed and analyzed using an auto-analyzer (Hitachi 747; Hitachi, Tokyo, Japan) at the central laboratory of the Kailuan General Hospital. Serum creatinine was measured with the enzymatic method in the 11 hospitals where participants were being followed. The coefficient of variation using blind quality control specimens was <2.0%. We calculated eGFR through modified 4-variable Chronic Kidney Disease Epidemiology Collaboration (CKD-EPI) formula and adjusted for the Chinese population by multiplying the coefficient 1.1:^21^ eGFR_CKD-EPI_ = 141 × min (SCr/κ,1)^α^ × max (SCr/κ,1)^−1.209^ × 0.993^Age^ × 1.018 (if female) × 1.1; SCr was serum creatinine, κ was 0.7 for females and 0.9 for males, α was −0.329 for females and −0.411 for males, min was the minimum of SCr/κ or 1, and max indicated the maximum of SCr/κ or 1. The eGFR values were divided into four levels: <45, 45 to 59, 60 to 89, ≥90 mL/min/1.73 m^2^, based on National Kidney Foundation’s Kidney Disease Outcomes Quality Initiative (NKFK/DOQI)^22^.

### Assessment of potential covariates

Demographic information (e.g. age, sex, smoking, and drinking) was collected via questionnaires, which were described previously^20^. Body mass index (BMI) is the ratio of body weight (kilograms) over height squared (square meters). Hypertension (HBP) was defined as systolic blood pressure ≥140 mmHg or diastolic blood pressure ≥90 mmHg, any use of antihypertensive drug, or self-reported history of hypertension. Diabetes mellitus (DM) was defined as fasting glucose level ≥7.0 mmol/L, non-fasting glucose concentration ≥11.1 mmol/L, any use of glucose-lowering drugs, or any self-reported history of diabetes. Dyslipidemia was defined as serum triglyceride ≥150 mg/dl, low density lipoprotein cholesterol ≥140 mg/dl, high density lipoprotein cholesterol ≤40 mg/dl, any use of lipid-lowering drugs, or any self-reported history of dyslipidemia.

### Assessment of ICAS

TCD was performed by two experienced neurologists using portable machines (Nicolet/EME Company, Germany). The two neurologists were blinded to the baseline information of the participants. ICAS was diagnosed mainly according to the peak systolic flow velocity (Vp) in terms of Wong’s published criterion^23^. Besides, participants’ age, presence of disturbance in echo frequency, turbulence, and the presence of segmental abnormality in velocity were also taken into consideration. ICAS was defined by a Vp > 140 cm/s for the middle cerebral artery; >120 cm/s for the anterior cerebral artery and internal carotid siphon; and >100 cm/s for the posterior cerebral artery and vertebra-basilar artery. The additional criterion of stenosis in MCA were: Vp ranged from 140 to 160 cm/s, together with disturbance in echo frequency and turbulence, or Vp reduction by ≥30% compared with the contralateral depth-corresponding homologous segment; or Vp > 160 cm/s regardless of disturbance in echo frequency and turbulence. For participants over 60 years old, the criteria mentioned above would lower 20 cm/s. In the absence of good temporal windows, intracranial blood flow signals were detected via orbital window. If certain arteries were failed to be detected via both temporal and orbital window, they were considered non-stenotic.

### Statistical analysis

Medians and interquartile ranges were used to describe quantitative variables, while numbers and proportions were used to describe enumeration variables. Normal distribution was checked using the Kolmogorov-Smirnoff test. As all the continuous variables in our analysis were non-normally distributed, so we used the Wilcoxon test to compare the differences between two groups and the Kruskal-Wallis test for comparison among multiple groups. The Chi-squared test was applied for the comparison of categorical variables and the non-parametric tests were used for ranked variables. Logistic regression models were performed to calculate the odds ratios (OR) and 95% confidence intervals (CI) for the associations of eGFR and proteinuria with asymptomatic ICAS. Model 1 adjusted for age and gender. Model 2 adjusted for age, gender, BMI, SBP, TC, TG, LDL cholesterol, HDL cholesterol, current smoking, current drinking, HBP, DM, dyslipidemia, education, income and physical activity. For each model analyzing the association between eGFR and asymptomatic ICAS, a trend test was performed after the eGFR level was entered into the model and treated as a continuous variable. Additionally, age and other potential indicators were also evaluated to assess if there was any significant interaction between these variables and the relationship between eGFR levels and asymptomatic ICAS presence. Two-sided P-values were reported for all the analyses. A P-value < 0.05 was considered to be statistically significant. All statistical analyses were performed by SAS software, version 9.3 (SAS Institute Inc., Cary, NC, USA).
